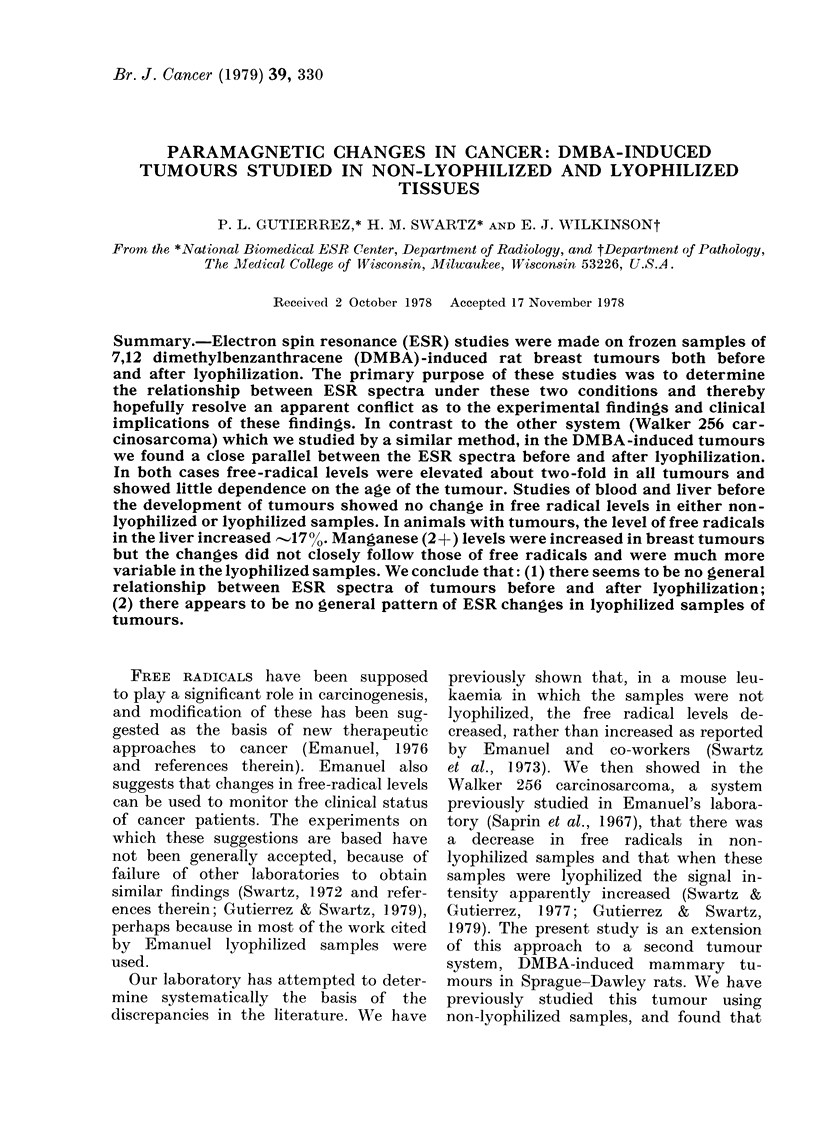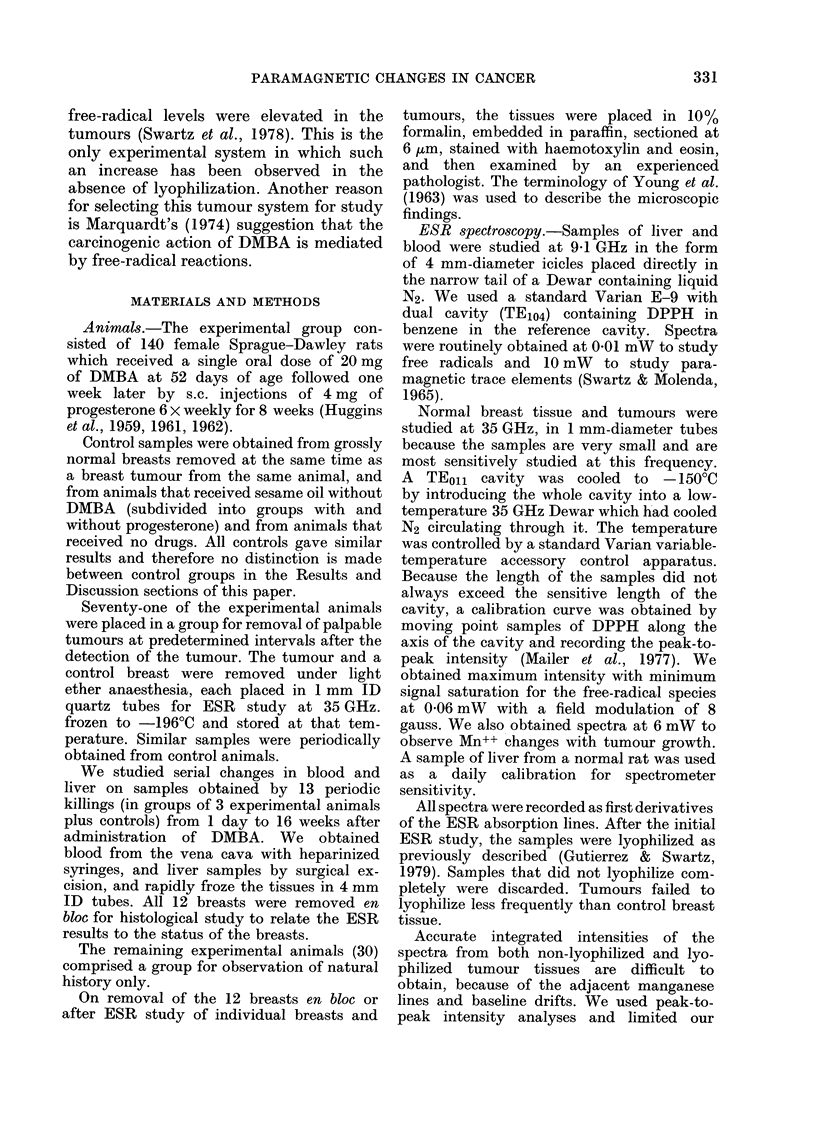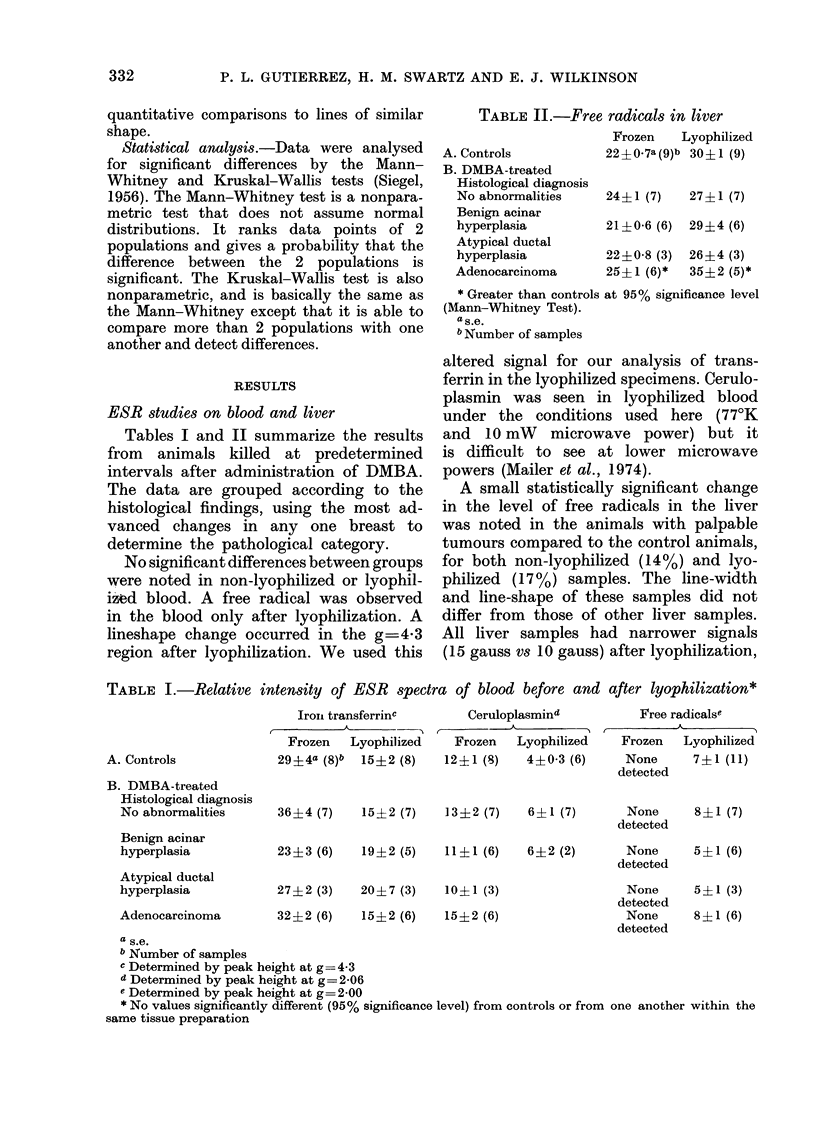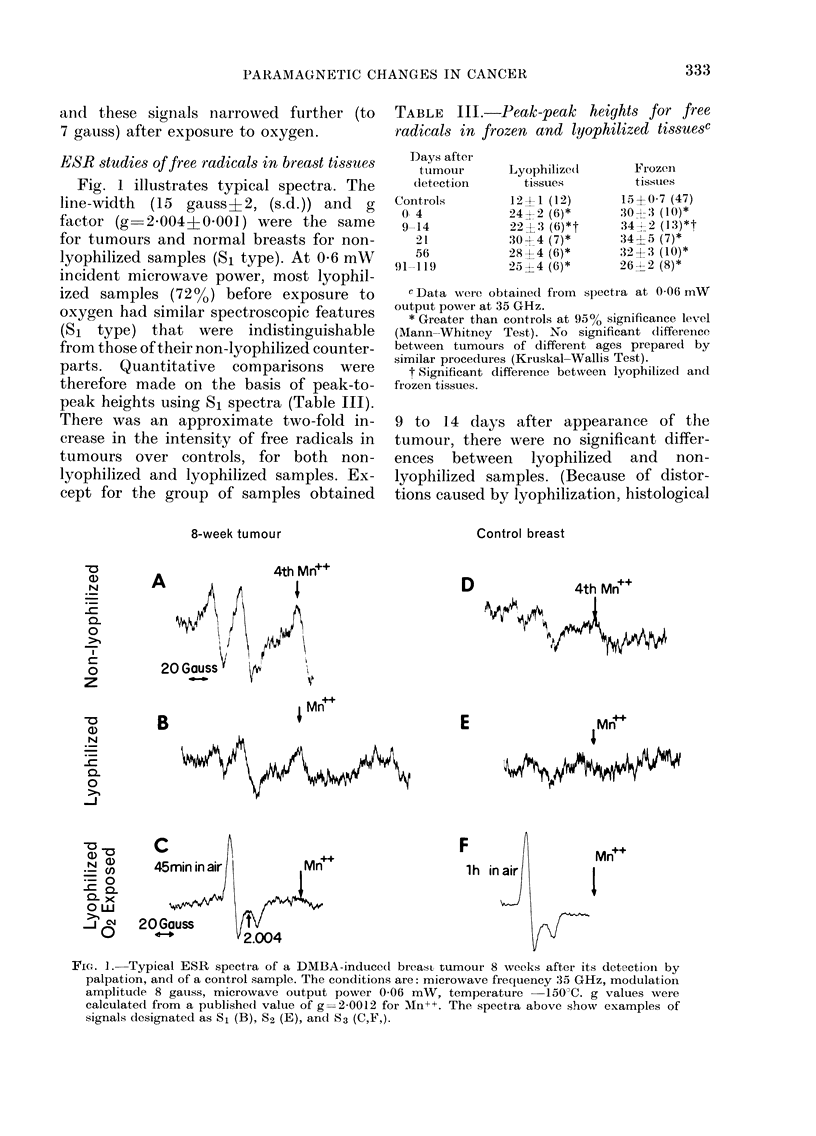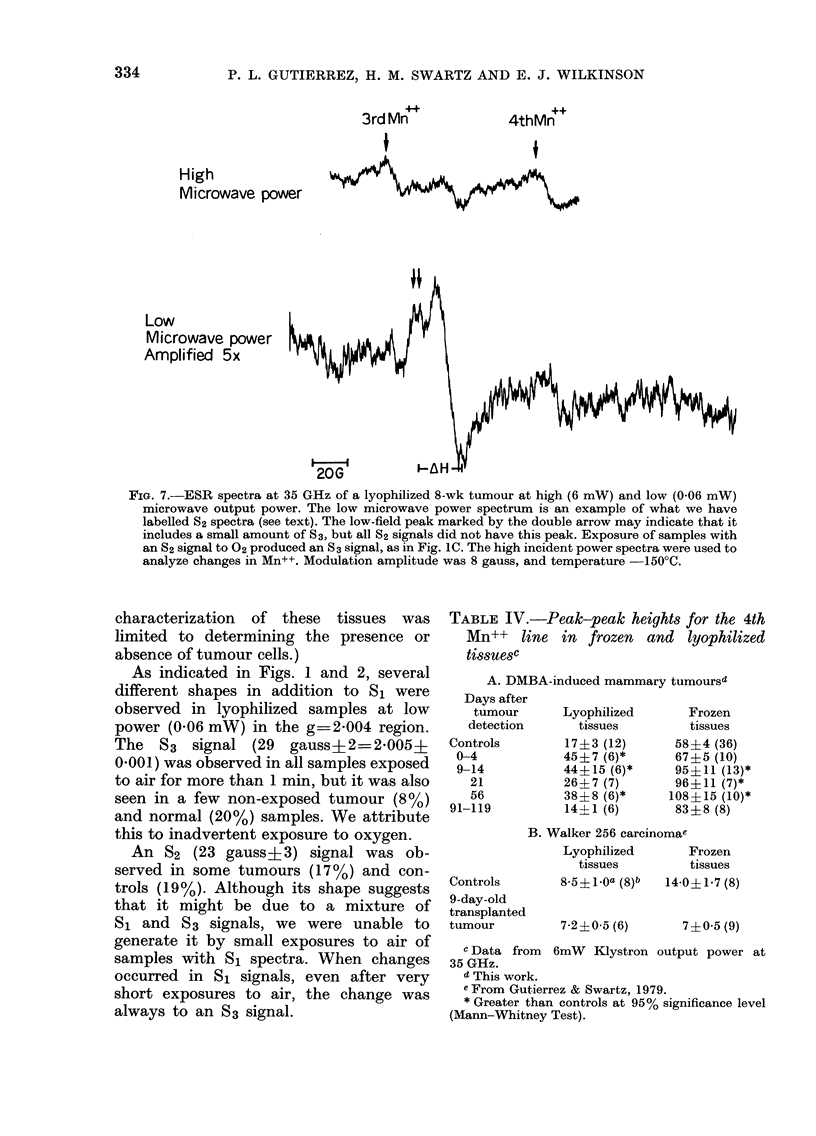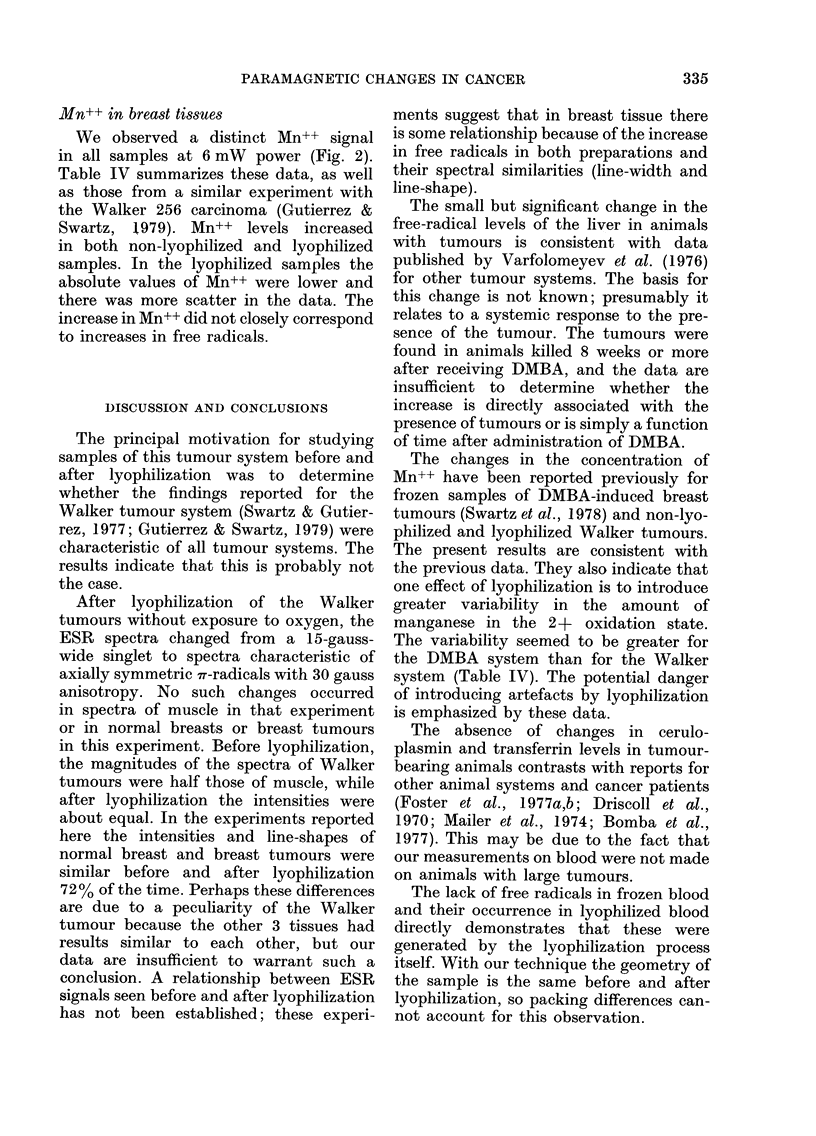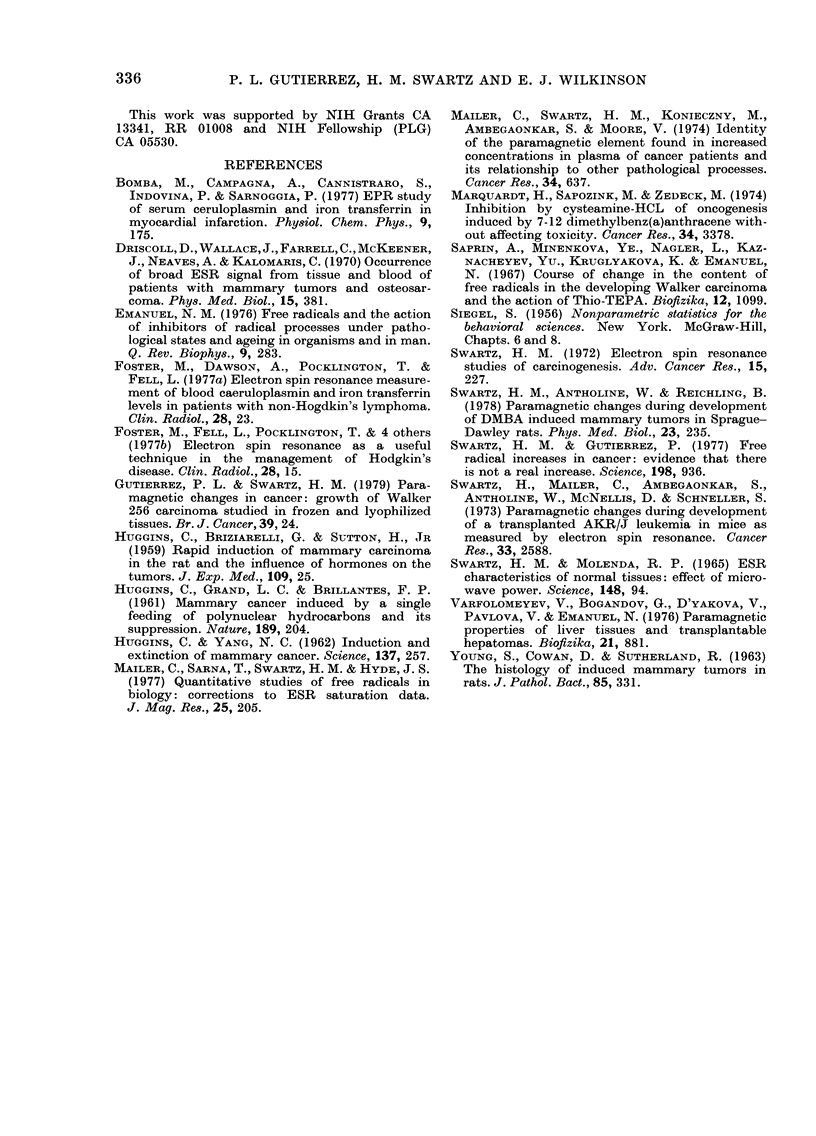# Paramagnetic changes in cancer: DMBA-induced tumours studied in non-lyophilized and lyophilized tissues.

**DOI:** 10.1038/bjc.1979.57

**Published:** 1979-03

**Authors:** P. L. Gutierrez, H. M. Swartz, E. J. Wilkinson

## Abstract

Electron spin resonance (ESR) studies were made on frozen samples of 7,12 dimethylbenzanthracene (DMBA)-induced rat breast tumours both before and after lyophilization. The primary purpose of these studies was to determine the relationship between ESR spectra under these two conditions and thereby hopefully resolve an apparent conflict as to the experimental findings and clinical implications of these findings. In contrast to the other system (Walker 256 carcinosarcoma) which we studied by a similar method, in the DMBA-induced tumours we found a close parallel between the ESR spectra before and after lyophilization. In both cases free-radical levels were elevated about two-fold in all tumours and showed little dependence on the age of the tumour. Studies of blood and liver before the development of tumours showed no change in free radicals levels in either nonlyophilized or lyophilized samples. In animals with tumours, the level of free radicals in the liver increased approximately 17%. Manganese (2+) levels were increased in breast tumours but the changes did not closely follow those of free radicals and were much more variable in the lyophilized samples. We conclude that: (1) there seems to be no general relationship between ESR spectra of tumours before and after lyophilization; (2) there appears to be no general pattern of ESR changes in lyophilized samples of tumours.


*Br. J. Cancer* (1979) **39**, 330

### PARAMAGNETIC CHANGES IN CANCER: DMBA-INDUCED TUMOURS STUDIED IN NON-LYOPHILIZED AND LYOPHILIZED TISSUES

P. L. GUTIERREZ,* H. M. SWARTZ* AND E. J. WILKINSON†

*From the *National Biomedical ESR Center, Department of Radiology, and †Department of Pathology, The Medical College of Wisconsin, Milwaukee, Wisconsin 53226, U.S.A.*

Received 2 October 1978 Accepted 17 November 1978

**Summary.—Electron spin resonance (ESR) studies were made on frozen samples of 7,12 dimethylbenzanthracene (DMBA)-induced rat breast tumours both before and after lyophilization. The primary purpose of these studies was to determine the relationship between ESR spectra under these two conditions and thereby hopefully resolve an apparent conflict as to the experimental findings and clinical implications of these findings. In contrast to the other system (Walker 256 carcinosarcoma) which we studied by a similar method, in the DMBA-induced tumours we found a close parallel between the ESR spectra before and after lyophilization. In both cases free-radical levels were elevated about two-fold in all tumours and showed little dependence on the age of the tumour. Studies of blood and liver before the development of tumours showed no change in free radical levels in either non-lyophilized or lyophilized samples. In animals with tumours, the level of free radicals in the liver increased $\sim$17%. Manganese (2+) levels were increased in breast tumours but the changes did not closely follow those of free radicals and were much more variable in the lyophilized samples. We conclude that: (1) there seems to be no general relationship between ESR spectra of tumours before and after lyophilization; (2) there appears to be no general pattern of ESR changes in lyophilized samples of tumours.**

FREE RADICALS have been supposed to play a significant role in carcinogenesis, and modification of these has been suggested as the basis of new therapeutic approaches to cancer (Emanuel, 1976 and references therein). Emanuel also suggests that changes in free-radical levels can be used to monitor the clinical status of cancer patients. The experiments on which these suggestions are based have not been generally accepted, because of failure of other laboratories to obtain similar findings (Swartz, 1972 and references therein; Gutierrez & Swartz, 1979), perhaps because in most of the work cited by Emanuel lyophilized samples were used.

Our laboratory has attempted to determine systematically the basis of the discrepancies in the literature. We have previously shown that, in a mouse leukaemia in which the samples were not lyophilized, the free radical levels decreased, rather than increased as reported by Emanuel and co-workers (Swartz *et al.*, 1973). We then showed in the Walker 256 carcinosarcoma, a system previously studied in Emanuel's laboratory (Saprin *et al.*, 1967), that there was a decrease in free radicals in non-lyophilized samples and that when these samples were lyophilized the signal intensity apparently increased (Swartz & Gutierrez, 1977; Gutierrez & Swartz, 1979). The present study is an extension of this approach to a second tumour system, DMBA-induced mammary tumours in Sprague–Dawley rats. We have previously studied this tumour using non-lyophilized samples, and found that free-radical levels were elevated in the tumours (Swartz *et al.*, 1978). This is the only experimental system in which such an increase has been observed in the absence of lyophilization. Another reason for selecting this tumour system for study is Marquardt's (1974) suggestion that the carcinogenic action of DMBA is mediated by free-radical reactions.

### MATERIALS AND METHODS

*Animals.*—The experimental group consisted of 140 female Sprague–Dawley rats which received a single oral dose of 20 mg of DMBA at 52 days of age followed one week later by s.c. injections of 4 mg of progesterone 6 × weekly for 8 weeks (Huggins *et al.*, 1959, 1961, 1962).

Control samples were obtained from grossly normal breasts removed at the same time as a breast tumour from the same animal, and from animals that received sesame oil without DMBA (subdivided into groups with and without progesterone) and from animals that received no drugs. All controls gave similar results and therefore no distinction is made between control groups in the Results and Discussion sections of this paper.

Seventy-one of the experimental animals were placed in a group for removal of palpable tumours at predetermined intervals after the detection of the tumour. The tumour and a control breast were removed under light ether anaesthesia, each placed in 1 mm ID quartz tubes for ESR study at 35 GHz. frozen to —196°C and stored at that temperature. Similar samples were periodically obtained from control animals.

We studied serial changes in blood and liver on samples obtained by 13 periodic killings (in groups of 3 experimental animals plus controls) from 1 day to 16 weeks after administration of DMBA. We obtained blood from the vena cava with heparinized syringes, and liver samples by surgical excision, and rapidly froze the tissues in 4 mm ID tubes. All 12 breasts were removed *en bloc* for histological study to relate the ESR results to the status of the breasts.

The remaining experimental animals (30) comprised a group for observation of natural history only.

On removal of the 12 breasts *en bloc* or after ESR study of individual breasts and tumours, the tissues were placed in 10% formalin, embedded in paraffin, sectioned at 6 $\mu$m, stained with haemotoxylin and eosin, and then examined by an experienced pathologist. The terminology of Young *et al.* (1963) was used to describe the microscopic findings.

*ESR spectroscopy.*—Samples of liver and blood were studied at 9·1 GHz in the form of 4 mm-diameter icicles placed directly in the narrow tail of a Dewar containing liquid $N_2$. We used a standard Varian E–9 with dual cavity ($TE_{104}$) containing DPPH in benzene in the reference cavity. Spectra were routinely obtained at 0·01 mW to study free radicals and 10 mW to study paramagnetic trace elements (Swartz & Molenda, 1965).

Normal breast tissue and tumours were studied at 35 GHz, in 1 mm-diameter tubes because the samples are very small and are most sensitively studied at this frequency. A $TE_{011}$ cavity was cooled to −150°C by introducing the whole cavity into a low-temperature 35 GHz Dewar which had cooled $N_2$ circulating through it. The temperature was controlled by a standard Varian variable-temperature accessory control apparatus. Because the length of the samples did not always exceed the sensitive length of the cavity, a calibration curve was obtained by moving point samples of DPPH along the axis of the cavity and recording the peak-to-peak intensity (Mailer *et al.*, 1977). We obtained maximum intensity with minimum signal saturation for the free-radical species at 0·06 mW with a field modulation of 8 gauss. We also obtained spectra at 6 mW to observe $Mn^{++}$ changes with tumour growth. A sample of liver from a normal rat was used as a daily calibration for spectrometer sensitivity.

All spectra were recorded as first derivatives of the ESR absorption lines. After the initial ESR study, the samples were lyophilized as previously described (Gutierrez & Swartz, 1979). Samples that did not lyophilize completely were discarded. Tumours failed to lyophilize less frequently than control breast tissue.

Accurate integrated intensities of the spectra from both non-lyophilized and lyophilized tumour tissues are difficult to obtain, because of the adjacent manganese lines and baseline drifts. We used peak-to-peak intensity analyses and limited our quantitative comparisons to lines of similar shape.

*Statistical analysis.*—Data were analysed for significant differences by the Mann–Whitney and Kruskal–Wallis tests (Siegel, 1956). The Mann–Whitney test is a nonparametric test that does not assume normal distributions. It ranks data points of 2 populations and gives a probability that the difference between the 2 populations is significant. The Kruskal–Wallis test is also nonparametric, and is basically the same as the Mann–Whitney except that it is able to compare more than 2 populations with one another and detect differences.

### RESULTS

### *ESR studies on blood and liver*

Tables I and II summarize the results from animals killed at predetermined intervals after administration of DMBA. The data are grouped according to the histological findings, using the most advanced changes in any one breast to determine the pathological category.

No significant differences between groups were noted in non-lyophilized or lyophilized blood. A free radical was observed in the blood only after lyophilization. A lineshape change occurred in the g=4·3 region after lyophilization. We used this altered signal for our analysis of transferrin in the lyophilized specimens. Ceruloplasmin was seen in lyophilized blood under the conditions used here (77°K and 10 mW microwave power) but it is difficult to see at lower microwave powers (Mailer *et al.*, 1974).

TABLE II.—*Free radicals in liver*

| | Frozen | Lyophilized |
|---|---|---|
| A. Controls | 22±0·7[a] (9)[b] | 30±1 (9) |
| B. DMBA-treated | | |
| Histological diagnosis | | |
| No abnormalities | 24±1 (7) | 27±1 (7) |
| Benign acinar hyperplasia | 21±0·6 (6) | 29±4 (6) |
| Atypical ductal hyperplasia | 22±0·8 (3) | 26±4 (3) |
| Adenocarcinoma | 25±1 (6)* | 35±2 (5)* |

* Greater than controls at 95% significance level (Mann–Whitney Test).
[a] s.e.
[b] Number of samples

A small statistically significant change in the level of free radicals in the liver was noted in the animals with palpable tumours compared to the control animals, for both non-lyophilized (14%) and lyophilized (17%) samples. The line-width and line-shape of these samples did not differ from those of other liver samples. All liver samples had narrower signals (15 gauss *vs* 10 gauss) after lyophilization,

TABLE I.—*Relative intensity of ESR spectra of blood before and after lyophilization**

| | Iron transferrin[c] | | Ceruloplasmin[d] | | Free radicals[e] | |
|---|---|---|---|---|---|---|
| | Frozen | Lyophilized | Frozen | Lyophilized | Frozen | Lyophilized |
| A. Controls | 29±4[a] (8)[b] | 15±2 (8) | 12±1 (8) | 4±0·3 (6) | None detected | 7±1 (11) |
| B. DMBA-treated | | | | | | |
| Histological diagnosis | | | | | | |
| No abnormalities | 36±4 (7) | 15±2 (7) | 13±2 (7) | 6±1 (7) | None detected | 8±1 (7) |
| Benign acinar hyperplasia | 23±3 (6) | 19±2 (5) | 11±1 (6) | 6±2 (2) | None detected | 5±1 (6) |
| Atypical ductal hyperplasia | 27±2 (3) | 20±7 (3) | 10±1 (3) | | None detected | 5±1 (3) |
| Adenocarcinoma | 32±2 (6) | 15±2 (6) | 15±2 (6) | | None detected | 8±1 (6) |

[a] s.e.
[b] Number of samples
[c] Determined by peak height at g=4·3
[d] Determined by peak height at g=2·06
[e] Determined by peak height at g=2·00
* No values significantly different (95% significance level) from controls or from one another within the same tissue preparation and these signals narrowed further (to 7 gauss) after exposure to oxygen.

*ESR studies of free radicals in breast tissues*

Fig. 1 illustrates typical spectra. The line-width (15 gauss±2, (s.d.)) and g factor (g=2·004±0·001) were the same for tumours and normal breasts for non-lyophilized samples ($S_1$ type). At 0·6 mW incident microwave power, most lyophilized samples (72%) before exposure to oxygen had similar spectroscopic features ($S_1$ type) that were indistinguishable from those of their non-lyophilized counterparts. Quantitative comparisons were therefore made on the basis of peak-to-peak heights using $S_1$ spectra (Table III). There was an approximate two-fold increase in the intensity of free radicals in tumours over controls, for both non-lyophilized and lyophilized samples. Except for the group of samples obtained 9 to 14 days after appearance of the tumour, there were no significant differences between lyophilized and non-lyophilized samples. (Because of distortions caused by lyophilization, histological

TABLE III.—*Peak-peak heights for free radicals in frozen and lyophilized tissues*[c]

| Days after tumour detection | Lyophilized tissues | Frozen tissues |
|---|---|---|
| Controls | 12±1 (12) | 15±0·7 (47) |
| 0–4 | 24±2 (6)* | 30±3 (10)* |
| 9–14 | 22±3 (6)*† | 34±2 (13)*† |
| 21 | 30±4 (7)* | 34±5 (7)* |
| 56 | 28±4 (6)* | 32±3 (10)* |
| 91–119 | 25±4 (6)* | 26±2 (8)* |

[c] Data were obtained from spectra at 0·06 mW output power at 35 GHz.

* Greater than controls at 95% significance level (Mann–Whitney Test). No significant difference between tumours of different ages prepared by similar procedures (Kruskal–Wallis Test).

† Significant difference between lyophilized and frozen tissues.

FIG. 1.—Typical ESR spectra of a DMBA-induced breast tumour 8 weeks after its detection by palpation, and of a control sample. The conditions are: microwave frequency 35 GHz, modulation amplitude 8 gauss, microwave output power 0·06 mW, temperature —150°C. g values were calculated from a published value of g=2·0012 for $Mn^{++}$. The spectra above show examples of signals designated as $S_1$ (B), $S_2$ (E), and $S_3$ (C,F,).

FIG. 7.—ESR spectra at 35 GHz of a lyophilized 8-wk tumour at high (6 mW) and low (0·06 mW) microwave output power. The low microwave power spectrum is an example of what we have labelled $S_2$ spectra (see text). The low-field peak marked by the double arrow may indicate that it includes a small amount of $S_3$, but all $S_2$ signals did not have this peak. Exposure of samples with an $S_2$ signal to $O_2$ produced an $S_3$ signal, as in Fig. 1C. The high incident power spectra were used to analyze changes in $Mn^{++}$. Modulation amplitude was 8 gauss, and temperature —150°C.

characterization of these tissues was limited to determining the presence or absence of tumour cells.)

As indicated in Figs. 1 and 2, several different shapes in addition to $S_1$ were observed in lyophilized samples at low power (0·06 mW) in the $g = 2{\cdot}004$ region. The $S_3$ signal (29 gauss$\pm 2 = 2{\cdot}005 \pm 0{\cdot}001$) was observed in all samples exposed to air for more than 1 min, but it was also seen in a few non-exposed tumour (8%) and normal (20%) samples. We attribute this to inadvertent exposure to oxygen.

An $S_2$ (23 gauss$\pm$3) signal was observed in some tumours (17%) and controls (19%). Although its shape suggests that it might be due to a mixture of $S_1$ and $S_3$ signals, we were unable to generate it by small exposures to air of samples with $S_1$ spectra. When changes occurred in $S_1$ signals, even after very short exposures to air, the change was always to an $S_3$ signal.

TABLE IV.—*Peak–peak heights for the 4th $Mn^{++}$ line in frozen and lyophilized tissues*[c]

A. DMBA-induced mammary tumours[d]

| Days after tumour detection | Lyophilized tissues | Frozen tissues |
|---|---|---|
| Controls | 17±3 (12) | 58±4 (36) |
| 0–4 | 45±7 (6)* | 67±5 (10) |
| 9–14 | 44±15 (6)* | 95±11 (13)* |
| 21 | 26±7 (7) | 96±11 (7)* |
| 56 | 38±8 (6)* | 108±15 (10)* |
| 91–119 | 14±1 (6) | 83±8 (8) |

B. Walker 256 carcinoma[e]

| | Lyophilized tissues | Frozen tissues |
|---|---|---|
| Controls | 8·5±1·0[a] (8)[b] | 14·0±1·7 (8) |
| 9-day-old transplanted tumour | 7·2±0·5 (6) | 7±0·5 (9) |

[c] Data from 6mW Klystron output power at 35 GHz.
[d] This work.
[e] From Gutierrez & Swartz, 1979.
* Greater than controls at 95% significance level (Mann–Whitney Test).

*$Mn^{++}$ in breast tissues*

We observed a distinct $Mn^{++}$ signal in all samples at 6 mW power (Fig. 2). Table IV summarizes these data, as well as those from a similar experiment with the Walker 256 carcinoma (Gutierrez & Swartz, 1979). $Mn^{++}$ levels increased in both non-lyophilized and lyophilized samples. In the lyophilized samples the absolute values of $Mn^{++}$ were lower and there was more scatter in the data. The increase in $Mn^{++}$ did not closely correspond to increases in free radicals.

### DISCUSSION AND CONCLUSIONS

The principal motivation for studying samples of this tumour system before and after lyophilization was to determine whether the findings reported for the Walker tumour system (Swartz & Gutierrez, 1977; Gutierrez & Swartz, 1979) were characteristic of all tumour systems. The results indicate that this is probably not the case.

After lyophilization of the Walker tumours without exposure to oxygen, the ESR spectra changed from a 15-gauss-wide singlet to spectra characteristic of axially symmetric $\pi$-radicals with 30 gauss anisotropy. No such changes occurred in spectra of muscle in that experiment or in normal breasts or breast tumours in this experiment. Before lyophilization, the magnitudes of the spectra of Walker tumours were half those of muscle, while after lyophilization the intensities were about equal. In the experiments reported here the intensities and line-shapes of normal breast and breast tumours were similar before and after lyophilization 72% of the time. Perhaps these differences are due to a peculiarity of the Walker tumour because the other 3 tissues had results similar to each other, but our data are insufficient to warrant such a conclusion. A relationship between ESR signals seen before and after lyophilization has not been established; these experiments suggest that in breast tissue there is some relationship because of the increase in free radicals in both preparations and their spectral similarities (line-width and line-shape).

The small but significant change in the free-radical levels of the liver in animals with tumours is consistent with data published by Varfolomeyev *et al.* (1976) for other tumour systems. The basis for this change is not known; presumably it relates to a systemic response to the presence of the tumour. The tumours were found in animals killed 8 weeks or more after receiving DMBA, and the data are insufficient to determine whether the increase is directly associated with the presence of tumours or is simply a function of time after administration of DMBA.

The changes in the concentration of $Mn^{++}$ have been reported previously for frozen samples of DMBA-induced breast tumours (Swartz *et al.*, 1978) and non-lyophilized and lyophilized Walker tumours. The present results are consistent with the previous data. They also indicate that one effect of lyophilization is to introduce greater variability in the amount of manganese in the 2+ oxidation state. The variability seemed to be greater for the DMBA system than for the Walker system (Table IV). The potential danger of introducing artefacts by lyophilization is emphasized by these data.

The absence of changes in ceruloplasmin and transferrin levels in tumour-bearing animals contrasts with reports for other animal systems and cancer patients (Foster *et al.*, 1977*a*,*b*; Driscoll *et al.*, 1970; Mailer *et al.*, 1974; Bomba *et al.*, 1977). This may be due to the fact that our measurements on blood were not made on animals with large tumours.

The lack of free radicals in frozen blood and their occurrence in lyophilized blood directly demonstrates that these were generated by the lyophilization process itself. With our technique the geometry of the sample is the same before and after lyophilization, so packing differences cannot account for this observation.

This work was supported by NIH Grants CA 13341, RR 01008 and NIH Fellowship (PLG) CA 05530.

### REFERENCES

BOMBA, M., CAMPAGNA, A., CANNISTRARO, S., INDOVINA, P. & SARNOGGIA, P. (1977) EPR study of serum ceruloplasmin and iron transferrin in myocardial infarction. *Physiol. Chem. Phys.*, **9**, 175.

DRISCOLL, D., WALLACE, J., FARRELL, C., MCKEENER, J., NEAVES, A. & KALOMARIS, C. (1970) Occurrence of broad ESR signal from tissue and blood of patients with mammary tumors and osteosarcoma. *Phys. Med. Biol.*, **15**, 381.

EMANUEL, N. M. (1976) Free radicals and the action of inhibitors of radical processes under pathological states and ageing in organisms and in man. *Q. Rev. Biophys.*, **9**, 283.

FOSTER, M., DAWSON, A., POCKLINGTON, T. & FELL, L. (1977*a*) Electron spin resonance measurement of blood caeruloplasmin and iron transferrin levels in patients with non-Hogdkin's lymphoma. *Clin. Radiol.*, **28**, 23.

FOSTER, M., FELL, L., POCKLINGTON, T. & 4 others (1977*b*) Electron spin resonance as a useful technique in the management of Hodgkin's disease. *Clin. Radiol.*, **28**, 15.

GUTIERREZ, P. L. & SWARTZ, H. M. (1979) Paramagnetic changes in cancer: growth of Walker 256 carcinoma studied in frozen and lyophilized tissues. *Br. J. Cancer*, **39**, 24.

HUGGINS, C., BRIZIARELLI, G. & SUTTON, H., JR (1959) Rapid induction of mammary carcinoma in the rat and the influence of hormones on the tumors. *J. Exp. Med.*, **109**, 25.

HUGGINS, C., GRAND, L. C. & BRILLANTES, F. P. (1961) Mammary cancer induced by a single feeding of polynuclear hydrocarbons and its suppression. *Nature*, **189**, 204.

HUGGINS, C. & YANG, N. C. (1962) Induction and extinction of mammary cancer. *Science*, **137**, 257.

MAILER, C., SARNA, T., SWARTZ, H. M. & HYDE, J. S. (1977) Quantitative studies of free radicals in biology: corrections to ESR saturation data. *J. Mag. Res.*, **25**, 205.

MAILER, C., SWARTZ, H. M., KONIECZNY, M., AMBEGAONKAR, S. & MOORE, V. (1974) Identity of the paramagnetic element found in increased concentrations in plasma of cancer patients and its relationship to other pathological processes. *Cancer Res.*, **34**, 637.

MARQUARDT, H., SAPOZINK, M. & ZEDECK, M. (1974) Inhibition by cysteamine-HCL of oncogenesis induced by 7-12 dimethylbenz(a)anthracene without affecting toxicity. *Cancer Res.*, **34**, 3378.

SAPRIN, A., MINENKOVA, YE., NAGLER, L., KAZNACHEYEV, YU., KRUGLYAKOVA, K. & EMANUEL, N. (1967) Course of change in the content of free radicals in the developing Walker carcinoma and the action of Thio-TEPA. *Biofizika*, **12**, 1099.

SIEGEL, S. (1956) *Nonparametric statistics for the behavioral sciences.* New York. McGraw-Hill, Chapts. 6 and 8.

SWARTZ, H. M. (1972) Electron spin resonance studies of carcinogenesis. *Adv. Cancer Res.*, **15**, 227.

SWARTZ, H. M., ANTHOLINE, W. & REICHLING, B. (1978) Paramagnetic changes during development of DMBA induced mammary tumors in Sprague–Dawley rats. *Phys. Med. Biol.*, **23**, 235.

SWARTZ, H. M. & GUTIERREZ, P. (1977) Free radical increases in cancer: evidence that there is not a real increase. *Science*, **198**, 936.

SWARTZ, H., MAILER, C., AMBEGAONKAR, S., ANTHOLINE, W., MCNELLIS, D. & SCHNELLER, S. (1973) Paramagnetic changes during development of a transplanted AKR/J leukemia in mice as measured by electron spin resonance. *Cancer Res.*, **33**, 2588.

SWARTZ, H. M. & MOLENDA, R. P. (1965) ESR characteristics of normal tissues: effect of microwave power. *Science*, **148**, 94.

VARFOLOMEYEV, V., BOGANDOV, G., D'YAKOVA, V., PAVLOVA, V. & EMANUEL, N. (1976) Paramagnetic properties of liver tissues and transplantable hepatomas. *Biofizika*, **21**, 881.

YOUNG, S., COWAN, D. & SUTHERLAND, R. (1963) The histology of induced mammary tumors in rats. *J. Pathol. Bact.*, **85**, 331.